# Collagen V Influences Homeostatic Maintenance of Patellar Tendon Failure Properties in Mature Female Mice

**DOI:** 10.1002/jor.70054

**Published:** 2025-08-25

**Authors:** Ryan J. Leiphart, Jeremy D. Eekhoff, Stephanie N. Weiss, Rebecca Betts, Jaclyn A. Carlson, Patrick L. Paglia‐Garcés, Nathaniel A. Dyment, David E. Birk, Louis J. Soslowsky

**Affiliations:** ^1^ McKay Orthopaedic Laboratory University of Pennsylvania Philadelphia Pennsylvania USA

**Keywords:** biomechanics, collagen V, extracellular matrix, homeostasis, tendon

## Abstract

The collagenous matrix of tendon provides mechanical integrity, allowing the tissue to withstand large tensile forces. While collagen I‐containing fibrils provide the major backbone of the tendon matrix, interactions with other collagen types are critical for matrix formation and maintenance. Of these less abundant collagens, collagen V regulates fibril nucleation and lateral growth. In previous work, mouse models with reduced collagen V production recapitulated the musculoskeletal complications of Classic Ehlers–Danlos Syndrome in tendon development and healing, demonstrating altered structure and inferior mechanical properties. However, the roles of collagen V in homeostasis of mature, healthy tendon remain unknown. Therefore, this study evaluated the role of collagen V in maintaining tendon homeostasis using inducible knockdown of *Col5a1*in mature mice. After 30 days of reduced collagen V expression, patellar tendons demonstrated changes consistent with impaired matrix remodeling. Extracellular matrix and matrix remodeling genes were differentially expressed, and the distribution of fibril diameters was significantly altered with reduced expression of collagen V. The functional consequence of collagen V knockdown was demonstrated using mechanical testing, which revealed a reduction in failure properties, although sub‐failure properties such as stiffness and modulus were not affected. Interestingly, differences between these results and prior studies on the impact of collagen V on development or healing suggest a distinct role for collagen V in maintaining the properties of mature, healthy tendon. In summary, this study demonstrated that collagen V is essential for homeostasis of adult tendons through maintenance of the collagenous matrix.

## 1 Introduction

While collagen I makes up the majority of the tendon extracellular matrix, other matrix constituents are also essential for establishing and maintaining functional tendon tissue. Collagen V is a regulatory fibrillar collagen which, despite relatively low abundance in the tendon extracellular matrix, has been shown to play a critical role in matrix regulation and tendon health. During fibrillogenesis, collagen V co‐assembles with collagen I to form heterotypic fibrils, where the NH_2_‐terminal domain of collagen V protrudes from the fibril surface in the gap region to interact with additional matrix constituents [1, 2]. In this process, collagen V regulates both initial fibril nucleation as well as lateral fibril growth [3, 4]. Systemic knockdown of *Col5a1* in mice is lethal by embryonic Day 10 with virtually no collagen fibril formation, illustrating the importance of collagen V for developing a functional extracellular matrix [5].

Mutations in *COL5A1* lead to tendon pathologies, including Classic Ehlers–Danlos Syndrome (cEDS) in humans with a prevalence of 1:20,000 people [6, 7, 8]. Patients with cEDS experience a wide range of orthopaedic complications, including joint hypermobility, which is likely caused by connective tissue dysregulation in the absence of collagen V [9, 10]. In agreement with this clinical complication, *Col5a1* ^*+/*^ *^−^* mice, a model for cEDS, were shown to have reduced tendon size and stiffness [11]. Tendon healing was also impaired in this model, which similarly matches observations of poor wound healing in cEDS patients [12, 13].

More recently, conditional and inducible genetic models that overcome the embryonic lethality of systemic *Col5a1* knockout mice have better defined the role of collagen V in tendon health. Tendon‐targeted knockout of *Col5a1* resulted in a dramatic phenotype with marked reductions in tendon size and mechanical properties, irregular fibril shape, and impaired limb function [14, 15, 16]. The use of inducible models to isolate the impact of collagen V in tendon healing demonstrated altered gene expression profiles and irregular fibril formation with *Col5a1* knockdown at the time of injury, although this surprisingly did not lead to mechanical deficits [17].

The regulatory role of collagen V in tendon homeostasis has not been distinguished from its role in development or its role in healing. Understanding this homeostatic role is critical for establishing the baseline effect of collagen V knockdown in both healthy and injured mature tendons. Therefore, the objective of this study was to determine the effect of acute knockdown of collagen V on the cellular, structural, and mechanical properties of mature, healthy tendons. Since the tendon fibril network is well‐established by tissue maturity [18], we hypothesized that acute knockdown of collagen V in mature tendons would result in minimal changes to tendon properties.

## 2 Methods

### 2.1 Animals

ROSA26‐CreERT2 (B6.129‐*Gt(ROSA)26Sor* ^*tm1(cre/ERT2)Tyj*^/J) mice were crossed with *Col5a1* floxed mice [19] to create ROSA26‐CreERT2;*Col5a1* ^*f/+*^ (I*‐Col5a1* ^*+/*^ *^−^*) and ROSA26‐CreERT2;*Col5a1* ^*f/f*^ (I‐*Col5a1^−^* ^*/*^ *^−^*) mice. (IACUC approved). At 120 days old (approximate human age equivalency: 20–30 years) [20], female mice received three consecutive daily tamoxifen (TM) injections (100 mg/kg body weight) to induce Cre‐mediated excision of floxed Col5a1 alleles, resulting in I‐*Col5a1* ^*+/*^ *^−^* and I‐*Col5a1^−/−^* genotypes which were compared to wild‐type (WT) controls, which also received tamoxifen injections at the same dose and timing. This study investigated female mice because of the greater incidence of EDS in females compared to males [21, 22]. Mice were killed 30 days post‐TM injections at 150 days old.

### 2.2 Gene Expression

At the time of sacrifice, right patellar tendons were isolated and immediately flash frozen at *−*80°C. After all samples were collected, patellar tendons were thawed in RNAlater ICE (Thermo, Waltham, MA), homogenized with plastic pestles in TRIzol, and further disrupted with vortexing. RNA was extracted (Direct‐zol RNA Microprep, Zymo, Irvine, CA), and cDNA was reverse‐transcribed (High‐Capacity cDNA RT, Thermo, Waltham, MA). cDNA was pre‐amplified for 15 cycles with Taqman assays for 48 target genes and was loaded into a Fluidigm 48.48 Dynamic Array (*n* = 6–8/group). The 48 target genes fit under broad categories of collagens, non‐collagenous matrix, matrix remodeling, differentiation/cell markers, and signaling and inflammation. *Abl1* and *Rps17* were used as housekeeper genes for the Fluidigm Array. Separate, RT‐qPCR reactions were performed to measure expression of *Col5a1* to determine knockdown efficiency with *Abl1* used as housekeeper (*n* = 5/group). For all data, ΔCT was calculated by subtracting the gene CT from the average housekeeping CT, and ΔΔCT was calculated by subtracting the gene ΔCT from the average WT ΔCT.

### 2.3 Histology

For histological analysis (*n* = 6/group), the knee joint was isolated by cutting through the femur and tibia at the time of sacrifice. The knee was flexed to 90°, placed into a cassette, fixed in formalin, and processed using standard paraffin histological techniques. Samples were embedded in paraffin, and sections were cut at 7 µm thickness before staining with hematoxylin and eosin. Three sections were imaged per sample. A 400 × 500 μm region of interest within the tendon midsubstance of each image was selected for analysis. Cells were automatically segmented using a custom MATLAB script. After manually selecting a region of interest to exclude non‐tendinous tissue and empty space, the number of cells normalized to the area of the region of interest and the median nuclear aspect ratio of all cells within the region of interest were determined. Reported values for each biological replicate are averaged across the three sections for that replicate.

### 2.4 Transmission Electron Microscopy

Transmission electron microscopy (TEM) was performed to determine collagen fibril diameter distributions (*n* = 4/group). Patellar tendon samples were fixed with Karnovsky's fixative and processed using standard procedures [23]. Post‐staining was performed using 2% aqueous uranyl acetate followed by 1% phosphotungstic acid to enhance contrast. Processed samples were sectioned at ~90 nm and imaged within the central portion of the tendon at 60,000× magnification using a JEOL 1400 TEM (JEOL, Tokyo, Japan). Ten regions were analyzed per tendon. Collagen fibril diameters were measured across the fibril minor axis for each image (BIOQUANT). Fibril diameter measurements were pooled across images to generate the distribution for each sample.

### 2.5 Biomechanics

Tibia‐patellar tendon‐patella complexes were harvested and finely dissected. The samples were cut into a dogbone shape by removing tissue on the lateral edges using a 2 mm biopsy punch. Tendon cross‐sectional area was measured, before and after cutting the dogbone shape, using a custom laser‐based device [24]. Tibias were secured using polymethyl methacrylate in custom pots, and patellas were secured in custom aluminum grips for mechanical testing.

Uniaxial tensile testing was performed on prepared samples (*n* = 15/group). The testing protocol consisted of 10 cycles of preconditioning, followed by stress relaxations at 3%, 4%, and 5% strain. Following each stress relaxation, frequency sweeps of 10 cycles at 0.1, 1, 5, and 10 Hz were performed. After the frequency sweep at 5% strain, samples returned to 0% strain and then were ramped to failure at 0.1% strain/second. Dynamic modulus (E*), and phase shift (δ) were quantified for each frequency sweep. Stiffness, modulus, maximum load, and maximum stress were quantified from the ramp to failure. Engineering stress and strain were used for all calculations.

Data from 14 samples were removed from analysis of the frequency sweep at 5% strain and the ramp to failure because the force at 5% strain exceeded the max force from the ramp to failure, indicating that damage occurred before the ramp to failure (4 WT, 5 I‐*Col5a1* ^*+/*^^−^, and 5 I‐*Col5a1* ^−*/*−^). Additionally, two samples were excluded from analysis of ramp to failure data due to loss of data from equipment malfunction (2 I‐*Col5a1* ^−*/*−^).

### 2.6 Statistics

All experiments and analyses were performed blinded. Normality of gene expression and biomechanics data were evaluated using Shapiro–Wilk tests. Gene expression, histological data, and biomechanics data were compared between genotypes using analysis of variance (ANOVA) with Tukey‐adjusted post‐hoc comparisons where appropriate. Collagen fibril diameter measurements were pooled across samples and distributions were compared between genotypes using Kolmogorov–Smirnov tests [25, 26, 27, 28]. Additionally, fibril diameter measurements were also binned in four quartiles within samples, and the relative frequency for each bin was compared across genotypes using ANOVA with Tukey‐adjusted post‐hoc comparisons where appropriate [29]. Descriptive statistics and statistical testing results for all experiments are included in Supporting Information S1: Tables 1–4.

## 3 Results

### 3.1 Knockdown of *Col5a1* Altered Expression of ECM and Matrix Remodeling Genes

**Figure 1:**
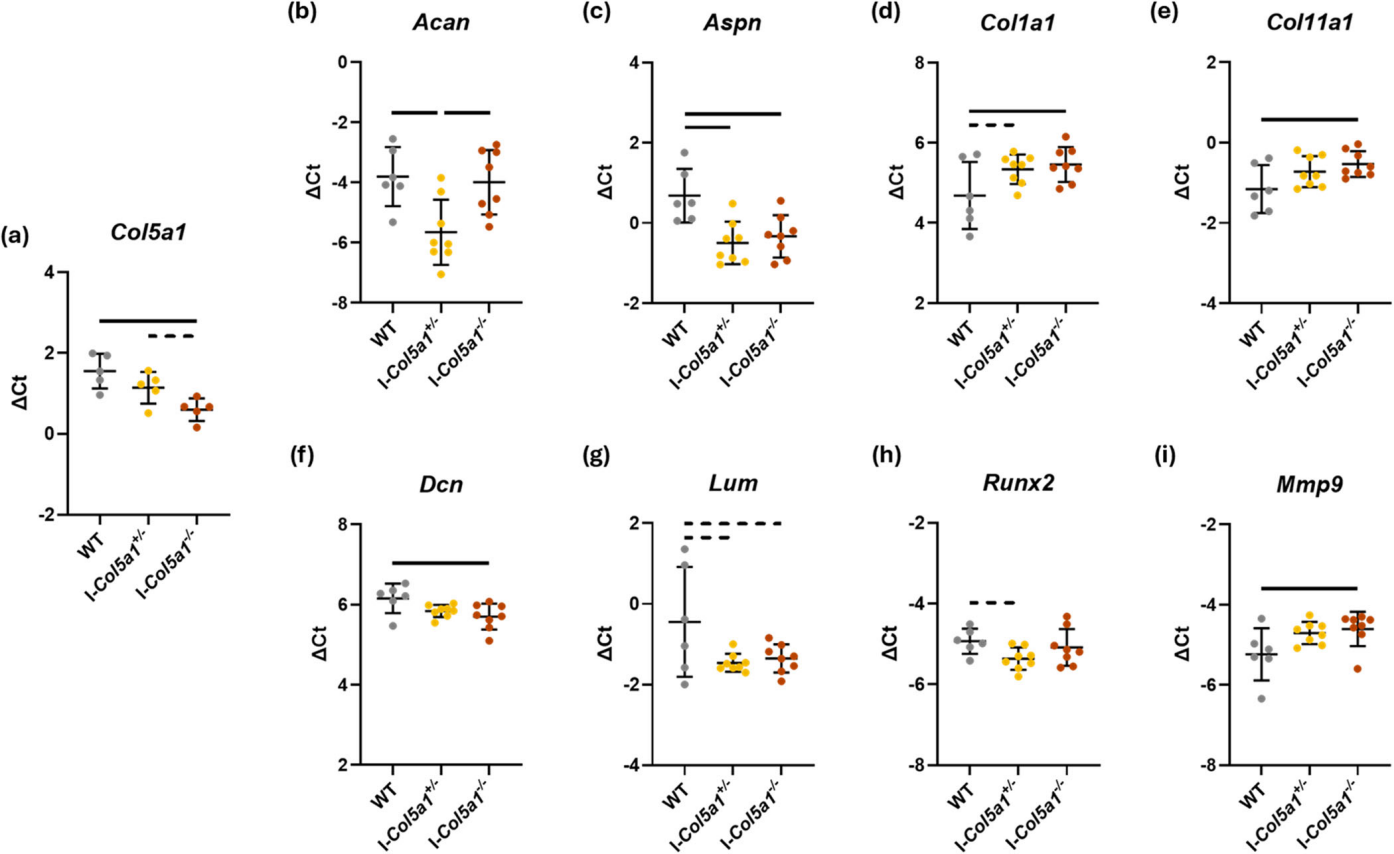
Collagen V knockdown impacted gene expression. (a) Tamoxifen induction significantly decreased *Col5a1* expression in I‐*Col5a1* ^−*/*−^ tendons. Expression of *Acan* (b), *Aspn* (c), *Col1a1* (d), *Col11a1* (e), *Dcn* (f), and *Mmp9* (i) was significantly affected by genotype. Differences in expression of *Lum* (g) and *Runx2* (h) were trending towards significance. Data shown as mean ± SD. Solid bars indicate *p* < 0.05, dashed bars indicate *p* < 0.1 from Tukey‐adjusted post‐hoc comparisons.

**Figure 2:**
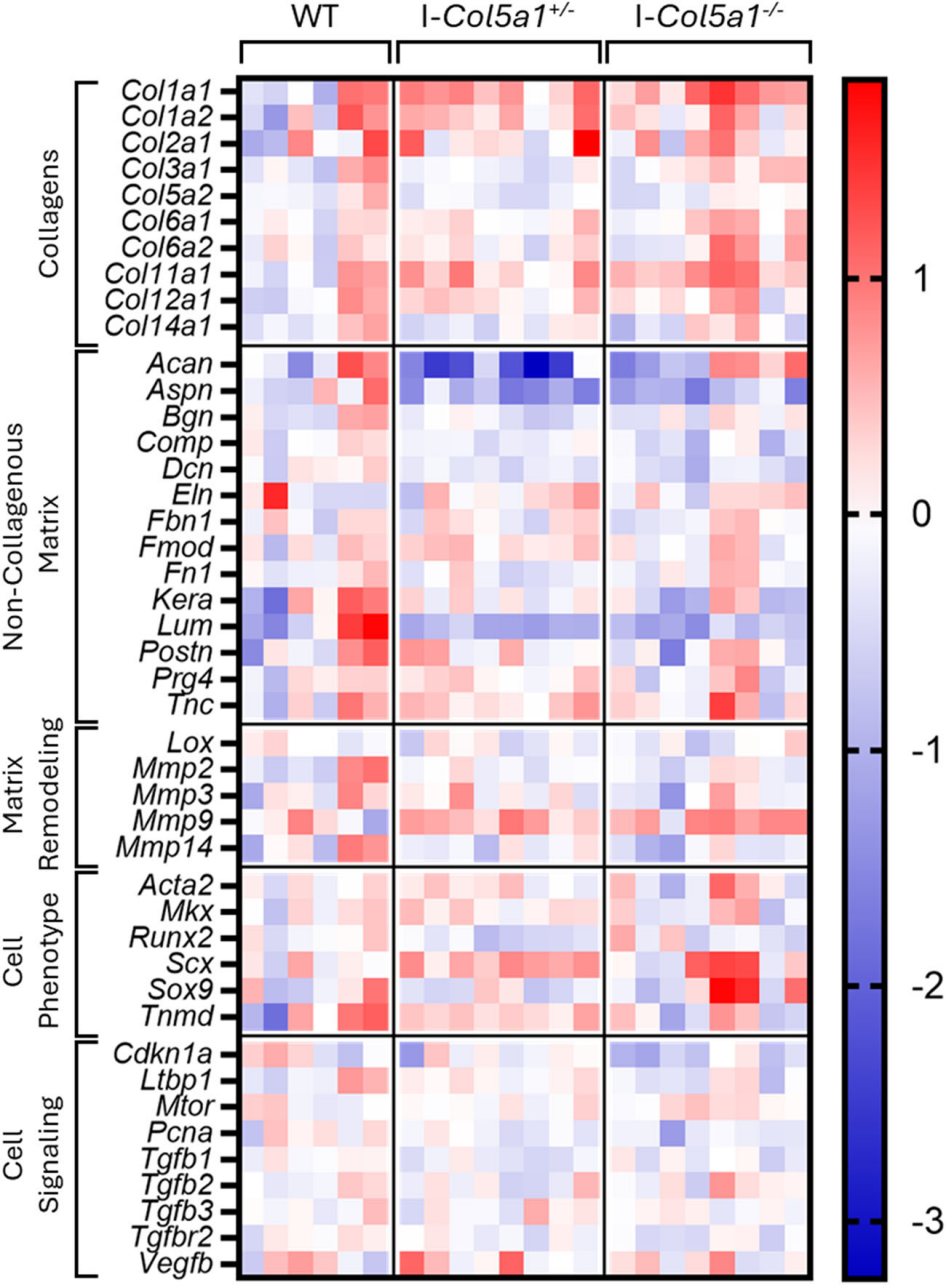
Heatmap visualization of the gene expression results from the Fluidigm Dynamic Array. Each element shows the ΔΔCT value for individual samples.

Tamoxifen induction of *Col5a1* knockdown decreased expression of *Col5a1* in I‐*Col5a1* ^−*/*−^ samples (Figure 1a). Expression of six genes from the Fluidigm array was significantly affected by genotype (Figure 2). *Acan* expression was decreased in I‐*Col5a* ^*+/*^^−^ tendons compared to WT and I‐*Col5a1* ^−*/*−^ (Figure 1b). *Aspn* expression was reduced in both I‐*Col5a1* ^*+/*^^−^ and I‐*Col5a1* ^−*/*−^ compared to WT (Figure 1c). Expression of *Col1a1*, *Col11a1*, and *Mmp9* expression was greater in I‐*Col5a1* ^−*/*−^ relative to WT tendons (Figure 1d,e,i), while *Dcn* expression was decreased in I‐*Col5a1* ^−*/*−^ compared to WT (Figure 1f). Two other genes, *Lum* and *Runx2*, had trending but nonsignificant differences in expression between genotypes (Figure 1g,h).

### 3.2 Reduction of *Col5a1* Expression did not Appreciably Change Tendon Morphology

**Figure 3:**
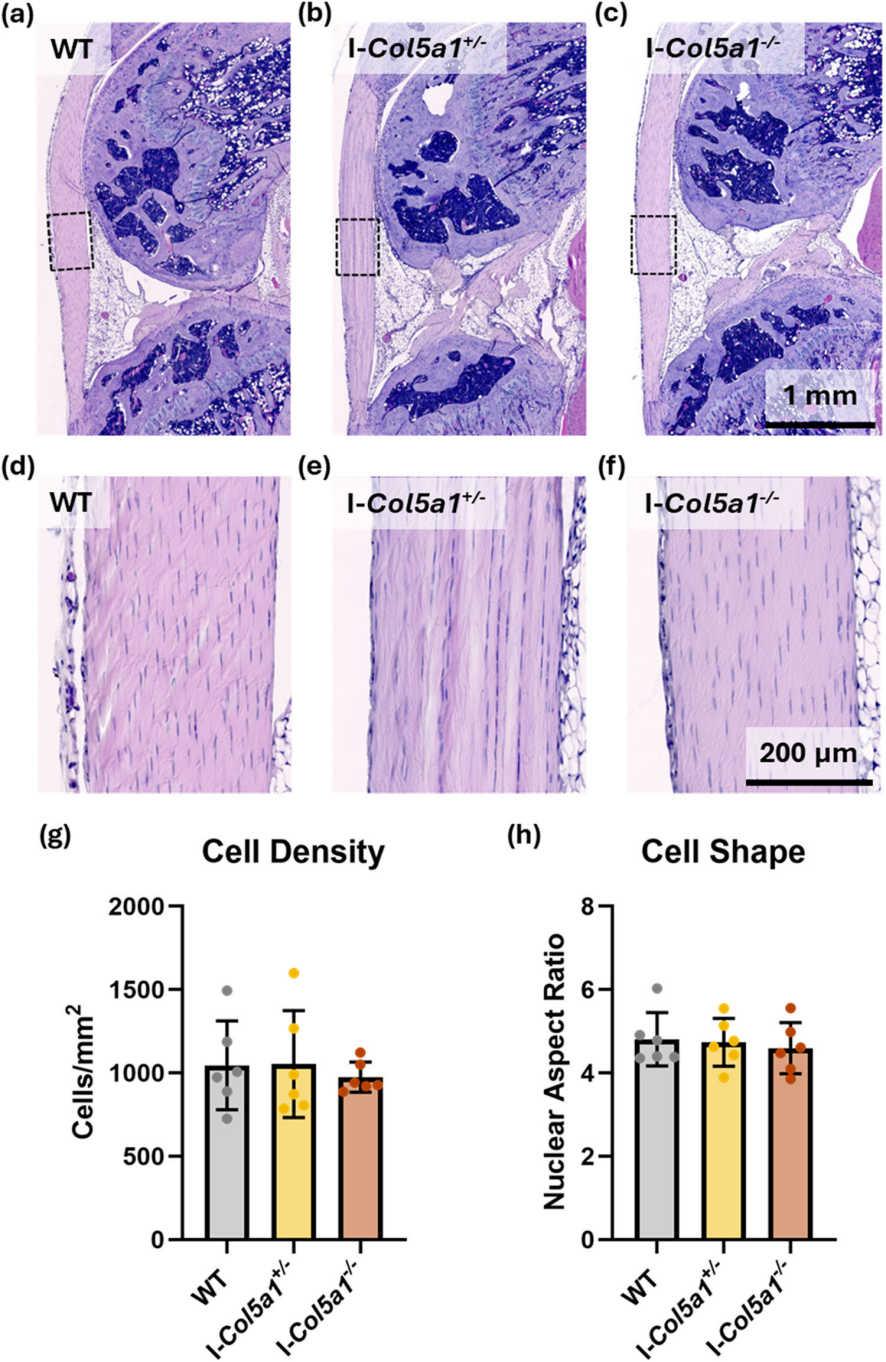
Histological appearance was unaffected by collagen V knockdown. Representative images of the full tendon (a–c) and selected ROI for analysis (d–f). Cell density (g) and cell shape (h) did not vary by genotype. Data shown as mean ± SD.

Morphology of the patellar tendon and surrounding tissues was assessed in H&E stained sections. Reduction of collagen V did not result in histological differences. Tendons from all genotypes appeared histologically normal, and there was no effect of genotype on either cellularity or cell shape (Figure 3). Similarly, there were no other appreciable changes in other structures in the knee.

### 3.3 Fibril Diameter Distributions Were Altered by *Col5a1* Knockdown

**Figure 4:**
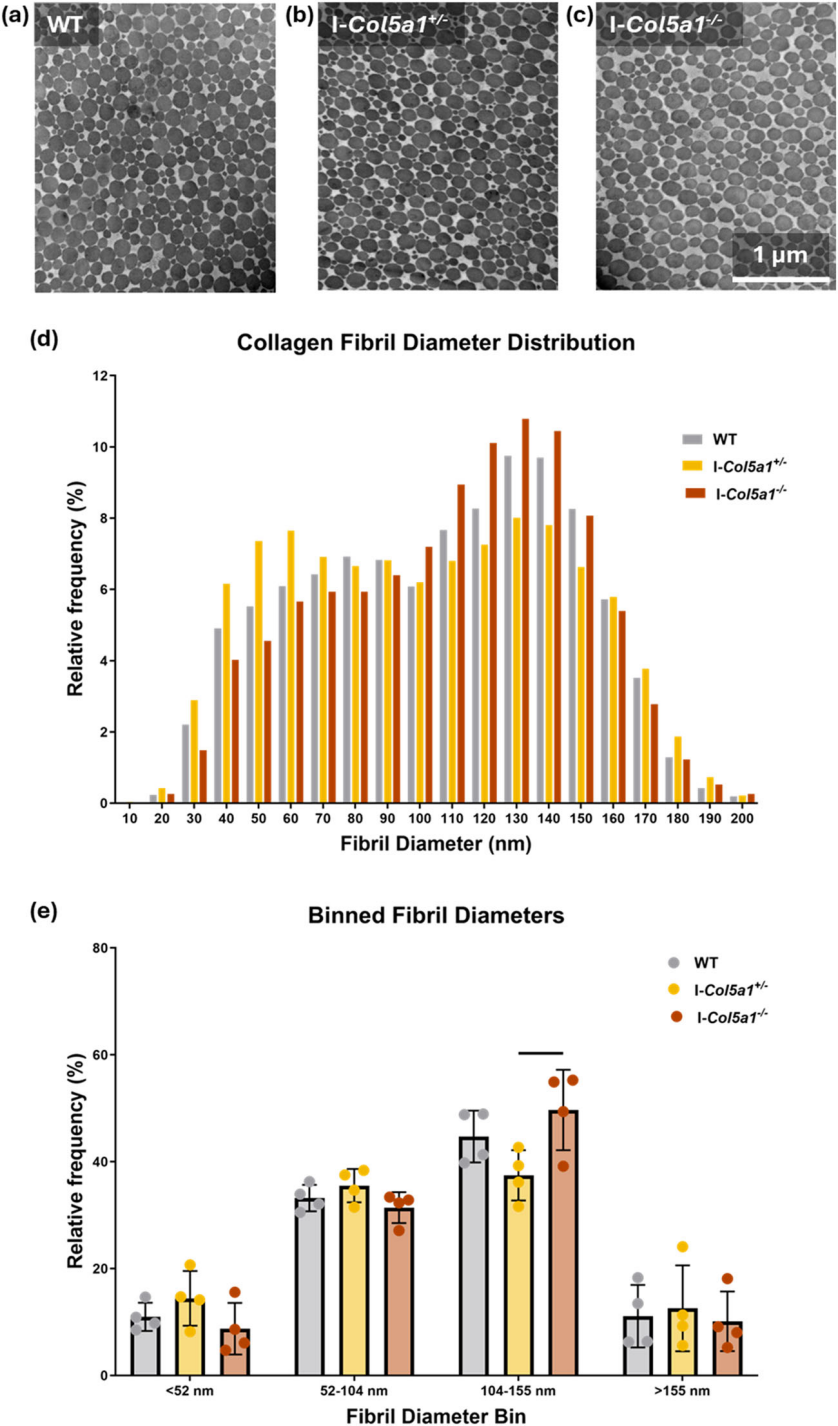
Collagen V knockdown altered collagen fibril diameter distributions. (a–c) Representative TEM images. (d) Fibril diameter distributions were significantly different between each genotype. (e) Binned analysis showed a difference in the relative frequency of fibrils with a diameter between 104 and 155 nm between I‐*Col5a1* ^*+/*^^−^ and I‐*Col5a1* ^−/−^ tendons. Solid bars indicate *p* < 0.05.

Fibril diameter distributions were significantly different (*p *< 0.001) between each genotype as determined using Kolmogorov–Smirnov tests (Figure 4). Binned analysis of fibril diameter measurements was then used to locate differences in the distributions. Tendons from I‐*Col5a1* ^−*/*−^ mice had a greater proportion of fibrils with diameters in the range of 104–155 nm compared to I‐*Col5a1* ^*+/*^^−^ tendons.

### 3.4 Failure Properties Were Reduced in *Col5a1* Knockdown Tendons

**Figure 5:**
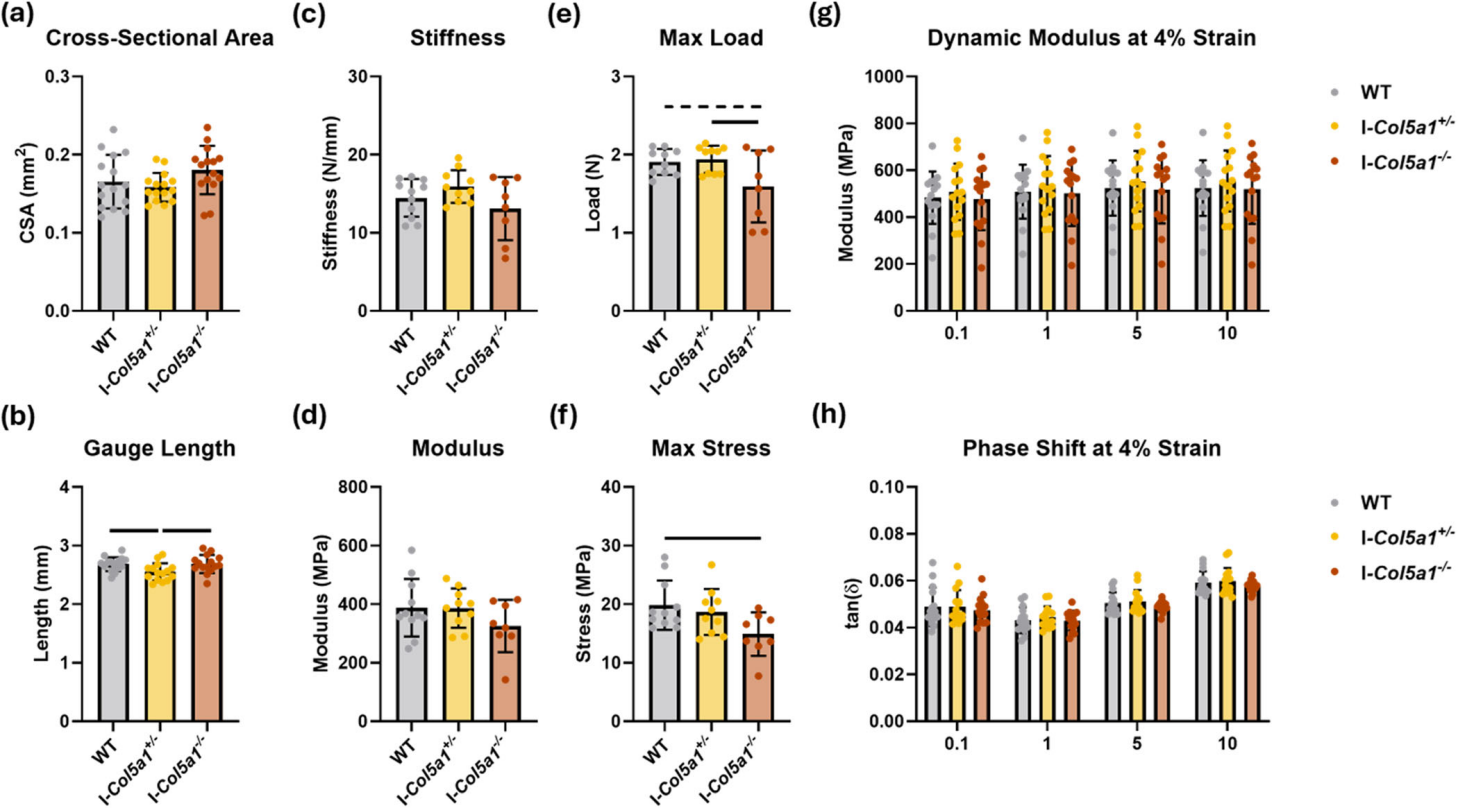
Collagen V knockdown reduced tendon failure properties. (a) Cross‐sectional area was unaffected by genotype. (b) Gauge length was subtly reduced in I‐*Col5a1* ^*+/*^^−^ tendons. Genotype did not affect stiffness (c) or modulus (d). Max load (e) and max stress (f) were reduced in I‐*Col5a1* ^−^^*/*^^−^ tendons. Dynamic modulus (g) and phase shift (f) did not vary by genotype. Data shown at 4% strain. Data at 3% and 5% strain are similarly unaffected by genotype (not shown). Data shown as mean ± SD. Solid bars indicate *p* < 0.05, dashed bars indicate *p* < 0.1 from Tukey‐adjusted post‐hoc comparisons.

Tendon cross‐sectional area was not affected by genotype (Figure 5a). Gauge length was decreased in I‐*Col5a1* ^*+/*^^−^ samples compared to WT and I‐*Col5a1* ^−^^*/*^^−^, indicating a slight reduction in tendon length (Figure 5b). While no changes in stiffness and modulus were detected with reduction of collagen V (Figure 5c,d), failure properties were affected by genotype. Max load was decreased in I‐*Col5a1* ^−^^*/*^^−^ compared to I‐*Col5a1* ^*+/*^^−^ tendons (Figure 5e), and max stress was decreased in I‐*Col5a1* ^−^^*/*^^−^ compared to WT (Figure 5f). All samples failed at the tibial insertion. However, there were no changes in dynamic modulus or phase shift at any strain or frequency (Figure 5g,h).

## 4 Discussion

Expanding on prior work investigating the roles of collagen V in tendon development and healing, this study evaluated how collagen V influences homeostasis of mature tendon using an inducible knockdown mouse model. Contrary to our hypothesis, knockdown of collagen V in mature tendon led to changes in tendon structure and function. Most notably, the mechanical strength of I‐*Col5a1* ^−^^*/*^^−^ tendons was impaired with reductions in both max load and max stress, indicating that collagen V is necessary to maintain failure properties in mature, healthy tendons. Concurrent changes to gene expression and tendon nanostructure may explain the observed mechanical changes.

Knockdown of *Col5a1* led to changes in the expression of extracellular matrix and matrix remodeling genes. The genes encoding small leucine‐rich proteoglycans (SLRPs) asporin and decorin were both downregulated with collagen V reduction, suggesting potential coordinate roles for SLRP interactions with collagens in maintaining the integrity of the collagen matrix [30, 31]. In contrast, *Acan* was only downregulated in I‐*Col5a1* ^*+/*^^−^ but not I‐*Col5a1* ^−^^*/*^^−^ tendons, which indicates a unique dose‐dependent response to collagen V knockdown that may preserve tendon function with a moderate reduction in *Col5a1* expression. Increased expression of *Mmp9* suggests increased matrix remodeling in response to collagen V knockdown. Upregulation of *Col1a1* and *Col11a1* similarly suggests increased remodeling, which may reflect compensatory mechanisms to maintain the integrity of the collagen matrix without collagen V. Notably, collagen XI has similarities in both structure and function to collagen V [32]. While collagen XI has typically been more closely associated with collagen II fibrils and collagen II‐rich tissue such as cartilage [33, 34], there is a growing body of literature demonstrating an important role for collagen XI in tendon [27, 35, 36]. However, upregulation of *Col11a1* was not sufficient to fully rescue the mechanical deficit caused by *Col5a1* knockdown in this model. Future work could continue to elucidate the synergistic roles of collagens V and XI in establishing and maintaining matrix architecture. Moreover, changes in other genes, such as *Lum* and *Runx2*, that did not reach statistical interest but may indicate subtle effects on the overall gene expression profile, may be another area of continued investigation.

Tissue‐level structural changes were less evident, though subtle changes to matrix nanostructure were observed. There were no histological abnormalities noted or any detected changes in cellularity or cell shape with collagen V knockdown. In contrast, genotype had a significant effect on the distribution of collagen fibril diameters, with an increase in the proportion of fibrils with diameters in the range of 104–155 nm in I‐*Col5a1* ^−^^*/*^^−^ compared to I‐*Col5a1* ^*+/*^^−^ tendons. While it is unclear how changes to fibril diameters impact tendon mechanics, the varying distributions further support remodeling of the collagen matrix occurring even within the short 30‐day window of this study.

The functional consequences of collagen V knockdown are evident in the changes to failure properties. Tendons from I‐*Col5a1* ^−^^*/*^^−^ failed at lower forces and lower stresses, indicating a meaningful reduction in mechanical integrity. Because the failure location was consistently at the tibial insertion, the change in failure properties may indicate a unique role for collagen V to regulate the matrix of the enthesis. However, sub‐failure properties were unaffected by knockdown, which contrasts with the reduction in stiffness that occurs when collagen V is absent during development [14]. Consequently, collagen V may have active yet distinct roles in tendon development and tendon homeostasis.

The mechanical deficit in I‐*Col5a1* ^−^^*/*^^−^ tendons after only 30 days of knockdown is surprising, since tendons have typically been described as having remarkably low rates of matrix turnover. For example, some studies describe minimal turnover of the tendon collagen matrix even over several decades [37, 38]. Even the shortest estimates of turnover times give a half‐life of 2 months [39, 40]. The turnover of collagen V in particular, while faster than collagen I, was projected to still take place over several months in rat tendon [41]. Interestingly, collagen V was shown to be in phase with collagen I throughout the circadian rhythm, which is critical for matrix homeostasis, and therefore, circadian control of tendon remodeling may be a relevant factor in our knockdown model [42]. Harmonizing this prior work on tendon matrix turnover with the results of the current study suggests that halting synthesis of new collagen V not only alters matrix remodeling but also accelerates it so that changes occur within a short period of time.

Isolating the effects of collagen V on homeostasis of mature tendon was made possible with this inducible knockdown mouse model. However, this model also carries with it the limitation of imperfect knockdown efficiency. We showed an average difference in ΔCT of 0.96 between WT and I‐*Col5a1* ^−^^*/*^^−^ tendons, which indicates *Col5a1* expression was reduced by 48% (i.e., 0.52‐fold change) after knockdown. Greater knockdown of *Col5a1* would have likely resulted in greater changes to tendon properties. Another limitation regarding knockdown efficiency is the lack of protein level quantification in this study. Future experiments could investigate changes in collagen V and other matrix proteins in this model. In addition, this study investigated only female mice, because some studies report a greater incidence of EDS in females compared to males [21, 22]. Previous work has shown sex‐dependent roles of collagen V in healing tendons, and future studies could evaluate how collagen V affects tendon homeostasis in males [12, 13]. Evaluating the long‐term effects of collagen V knockdown in mature tendon could also be an interesting area of continued research.

In summary, this study demonstrated that continued synthesis of collagen V is necessary for homeostasis of the mature tendon. Knockdown of collagen V altered fibril populations and reduced failure properties of the patellar tendon, likely through impaired and accelerated matrix remodeling. These results may ultimately inform strategies to improve management of cEDS in adult patients.

## Author Contributions

**Ryan J. Leiphart:** methodology, data acquisition, and data analysis. **Jeremy D. Eekhoff:** data analysis, interpretation, and manuscript writing. **Stephanie N. Weiss:** mouse colony maintenance and injections. **Rebecca Betts:** data acquisition. **Jaclyn A. Carlson:** data acquisition. Patrick L. Paglia‐Garcés: data acquisition. **Nathaniel A. Dyment:** methodology and supervision. **David E. Birk:** conceptualization and supervision. **Louis J. Soslowsky:** conceptualization, supervision.

## Supporting information


**Supplemental Table 1:** Descriptive statistics and *p*‐values for gene expression. **Supplemental Table 2:** Descriptive statistics and *p*‐values for histological data. **Supplemental Table 3:** Descriptive statistics and *p*‐values for fibril diameter distributions. **Supplemental Table 4:** Descriptive statistics and *p*‐values for binned fibril diameter analysis. **Supplemental Table 5:** Descriptive statistics and *p*‐values for mechanical properties.
