# Supplementary material for: Collagen V Influences Homeostatic Maintenance of Patellar Tendon Failure Properties in Mature Female Mice
Source: J Orthop Res. 2025 Aug 25;44(1):e70054. doi: 10.1002/jor.70054 (PMC12701723; doi:10.1002/jor.70054)
Supplement: Supplementary file 1 — Supplemental Table 1: Descriptive statistics and p‐values for gene expression. Supplemental Table 2: Descriptive statistics and p‐values for histological data. Supplemental Table 3: Descriptive statistics and p‐values for fibril diameter distributions. Supplemental Table 4: Descriptive statistics and p‐values for binned fibril diameter analysis. Supplemental Table 5: Descriptive statistics and p‐values for mechanical properties. [file JOR-44-0-s001.docx]

**Supplemental Table 1.** Descriptive statistics and p-values for gene expression.

**Supplemental Table 2.** Descriptive statistics and p-values for histological data.

**Supplemental Table 3.** Descriptive statistics and p-values for fibril diameter distributions.

**Supplemental Table 4.** Descriptive statistics and p-values for binned fibril diameter analysis.

**Supplemental Table 5.** Descriptive statistics and p-values for mechanical properties.
